# Intraspecific priority effects in response to egg hatching delay in a pond-breeding salamander

**DOI:** 10.1038/s41598-024-70140-z

**Published:** 2024-08-21

**Authors:** Thomas L. Anderson, Trevor J. Rallo

**Affiliations:** Department of Biological Sciences, Southern Illinois University Edwardsville, Box 1651, Edwardsville, IL 62026 USA

**Keywords:** Amphibian, Ambystoma, Cannibalism, Phenology, Seasonality, Thinning effect, Climate-change ecology, Freshwater ecology

## Abstract

As reproduction phenologies shift with climate change, populations can experience intraspecific priority effects, wherein early hatching cohorts experience an advantage over late-hatching cohorts, resulting in altered demography. Our study objective was to identify how variation in egg hatching phenology alters intraspecific interactions in small-mouthed salamanders, *Ambystoma texanum.* We addressed two research questions: (Q1) How are demographic responses altered by variation in the temporal duration of hatching between cohorts, and (Q2) How does the seasonality of hatching delays affect demographic responses? We manipulated hatching phenologies of *A. texanum* eggs and reared larvae in outdoor mesocosms to metamorphosis. For Q1, hatching delay exhibited non-linear relationships with survival and body size, with the greatest asynchrony in cohort additions resulting in the highest mortality and largest body sizes. For Q2, hatching delay effects were stronger (i.e., survival was lower and body sizes larger) when they occurred later in the season, potentially due to temperature differences that larvae experienced. Overall, our results demonstrate that changes in intraspecific interactions due to phenological shifts can be context-dependent, depending on the strength (i.e., temporal duration) and seasonality of such processes. Identifying context-dependencies of phenological shifts will be critical for predicting changes in organismal demographics with climatic shifts.

## Introduction

One of the most conspicuous impacts of climate change that is affecting taxa worldwide is shifts in the timing of biological events (i.e., phenology)^1,2^. An expected consequence of phenological shifts is altered demography and population dynamics^3,4^. For example, organisms may experience novel environmental conditions as their phenology shifts, leading to altered vital rates^5^. Additionally, population demographics may change due to altered species interactions that occur due to greater timing (a)synchronies among species^6^. The exact nature of how phenological shifts will alter population demographics is still uncertain for many taxa, however, making mechanistic tests of phenological change crucial to understand why and how much demography may be affected.

One mechanism behind demographic changes due to phenological shifts is skewed offspring body size distributions, generated by altered patterns of adult breeding phenology. In this situation, size advantages gained by offspring of early-arriving individuals facilitates their superiority in antagonistic interactions over offspring of later-arriving individuals, i.e. size-mediated priority effects (SMPE]^7^. The degree to which early-arriving individuals gain a size-based advantage is contingent on a variety of conditions, such as the suitability of growing conditions at each cohorts’ arrival or the time duration between arrival of each cohort. Thus, the biotic and abiotic environments can interact to shape the relative magnitude or importance of priority effects and SMPE specifically^8^.

While SMPE have been documented in a variety of taxa, including fish^9^ and insects^7,10,11^, they are especially prevalent in pond-breeding amphibians^12–14^. They often occur within this group due to the commonality of hatching asynchronies among individuals within a population or across species, which has led to this group being a model system for understanding priority effects more generally for more than four decades^15–18^. In particular, previous experimental research has shown that SMPE can substantially affect demographic rates of aquatic stages^12,14,19–21^. However, the majority of work on SMPE has examined its outcomes under a limited set of conditions. For example, the range of temporal differences in phenology used are often limited, e.g. individuals added simultaneously vs two cohorts added on distinct days^20,21^. Yet breeding phenology is highly variable across populations and years due to differences in weather that dictates adult breeding migrations^22,23^. This variation in weather can lead to differences in the degree to which populations are synchronized in their breeding efforts (i.e., variable numbers of breeding pulses or durations of spacing between breeding pulses). Furthermore, the relative timing of breeding within a season (e.g., overall population shifts to early or later breeding) can result in different thermal conditions developing eggs and larvae of all cohorts subsequently experience, potentially leading to differences in outcomes of species interactions based on temperature^13,24^. Investigation of such nuanced details can help inform the degree to which population demographics may be altered in response to phenological shifts.

The goal of this study was to examine how variation in the timing of hatching influenced SMPEs in the small-mouthed salamander, *Ambystoma texanum*. We manipulated hatching phenologies in the laboratory, prior to rearing salamanders in outdoor mesocosms (tanks) until metamorphosis. Using these methodologies, we examined two specific research questions within our overarching goal: (Q1) How do phenotypic traits of individuals (body size and development rates) and survival shift with increasing differences in the timing of hatching among cohorts (hereafter, hatching delay), and (Q2) How does the timing of when hatching delays occur in the season (early or late) affect demographic responses? We expected that increasing hatching delay (greater temporal separation of entry into ponds) would lead to greater interference competition and potentially cannibalism from earlier cohorts on later cohorts, i.e. stronger SMPEs. We also predicted that when breeding occurs later in the season, SMPEs would be stronger due to warmer water temperatures allowing for greater individual growth rates of early arrivers that would lead to greater body size disparities. In both situations, if hatching delays affected overall survival, we expected body size and development rates to increase due to reduce competition among survivors, i.e., thinning effects^25^.

## Results

### Question 1: Hatching delay

**Figure 1:**
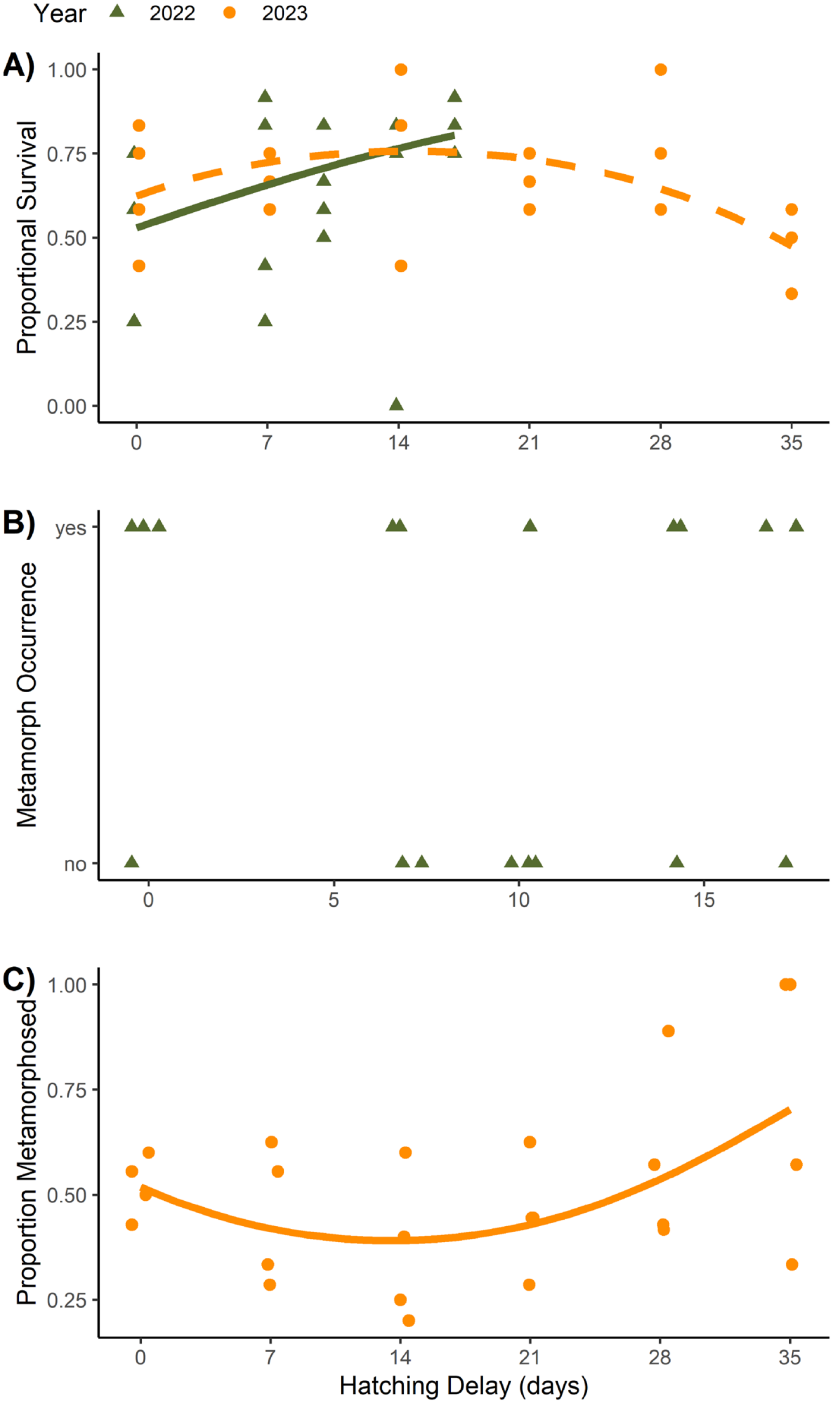
Survival (**A**), the occurrence of metamorphosis in 2022 (**B**) and proportion metamorphosed in 2023 (**C**) in response to hatching delay treatments for *A. texanum*. Each circle represents one tank. Points are adjusted horizontally in to minimize overlap. Note the different x-axis scale in (**B**). Relationships were statistically significant in (**A**) and (**C**), but not (**B**).

Survival was generally high in most tanks, with 65% of all animals recovered across both years. Linear and quadratic models of survival were not different based on likelihood ratio tests (LRT) in 2022 (χ^2^ = 2.69, *P* = 0.10), so we assessed the more parsimonious linear model. Hatching delay was positively and significantly related to survival (Table 1, Fig. 1a). All replicates had survival values that exceeded 75% in the 17-d difference in addition (Fig. 1a). In contrast, survival was more variable when all hatchlings were added on the same day (Fig. 1a). In 2023, survival was nonlinearly related to hatching delay, based on a significant LRT between linear and quadratic models (χ^2^ = 8.34, *P* = 0.004). Survival again peaked around a 14-day delay, with a large drop-off in survival between the 28-d and 35-d delay treatments (Fig. 1a).

**Table 1:** Parameter estimates of final generalized linear models for survival and metamorphosis in Q1. Survival indicates the number of individuals recovered (larvae + metamorphs). Metamorphosis in 2022 is the presence of any metamorphs in a tank, while in 2023 this response was the proportion of survivors having undergone metamorphosis. For parameters, Delay is the linear coefficient associated with the hatching delay treatment, and Duration2 is the quadratic coefficient associated with hatching delay treatment.

Year	Response	Parameter	Estimate	SE	*P* value
2022	Survival	Intercept	0.168	0.251	0.504
		Delay	0.076	0.025	0.030
	Metamorphosis	Intercept	0.302	0.867	0.728
		Delay	0.011	0.140	0.889
2023	Survival	Intercept	0.506	0.273	0.064
		Delay	0.086	0.038	0.022
		Delay2	− 0.003	0.001	0.004
	Metamorphosis	Intercept	0.071	0.326	0.828
		Delay	− 0.076	0.045	0.089
		Delay2	0.003	0.001	0.029

Differences in experimental duration resulted in analysis of different metrics of completion of metamorphosis across years (see Methods). Metamorphosis of at least one individual was recorded in 12 of the 20 tanks in 2022. The linear model was more supported than the quadratic model (LRT: χ^2^ = 2.23, *P* = 0.13), but hatching was statistically unrelated to hatching delay (Table 1; Fig. 1b). The proportion of survivors completing metamorphosis in 2023 was nonlinearly related to hatching delay (LRT between linear and quadratic models: χ^2^ = 4.91, *P* = 0.03). Metamorphosis rates of survivors significantly increased with greater delay, especially between the 28-d and 35-d treatments (Fig. 1c).

**Figure 2:**
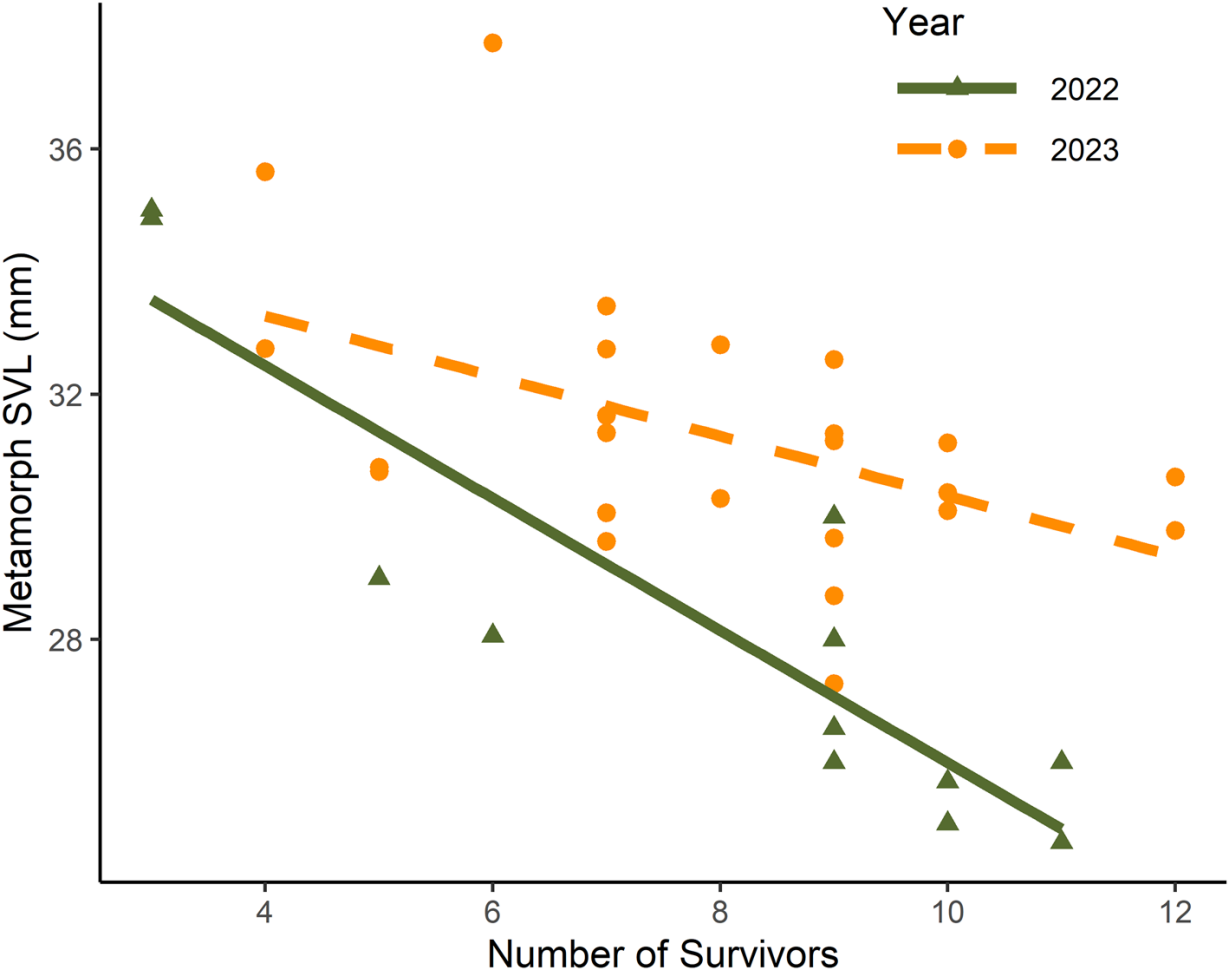
Snout-vent length (SVL, in mm) of metamorphs in response to total number of survivors in a tank. Each point represents a tank average. Point shape, color and line type indicates year. Models were fit separately for each year, so the crossing lines do not represent a true statistical interaction.

**Figure 3:**
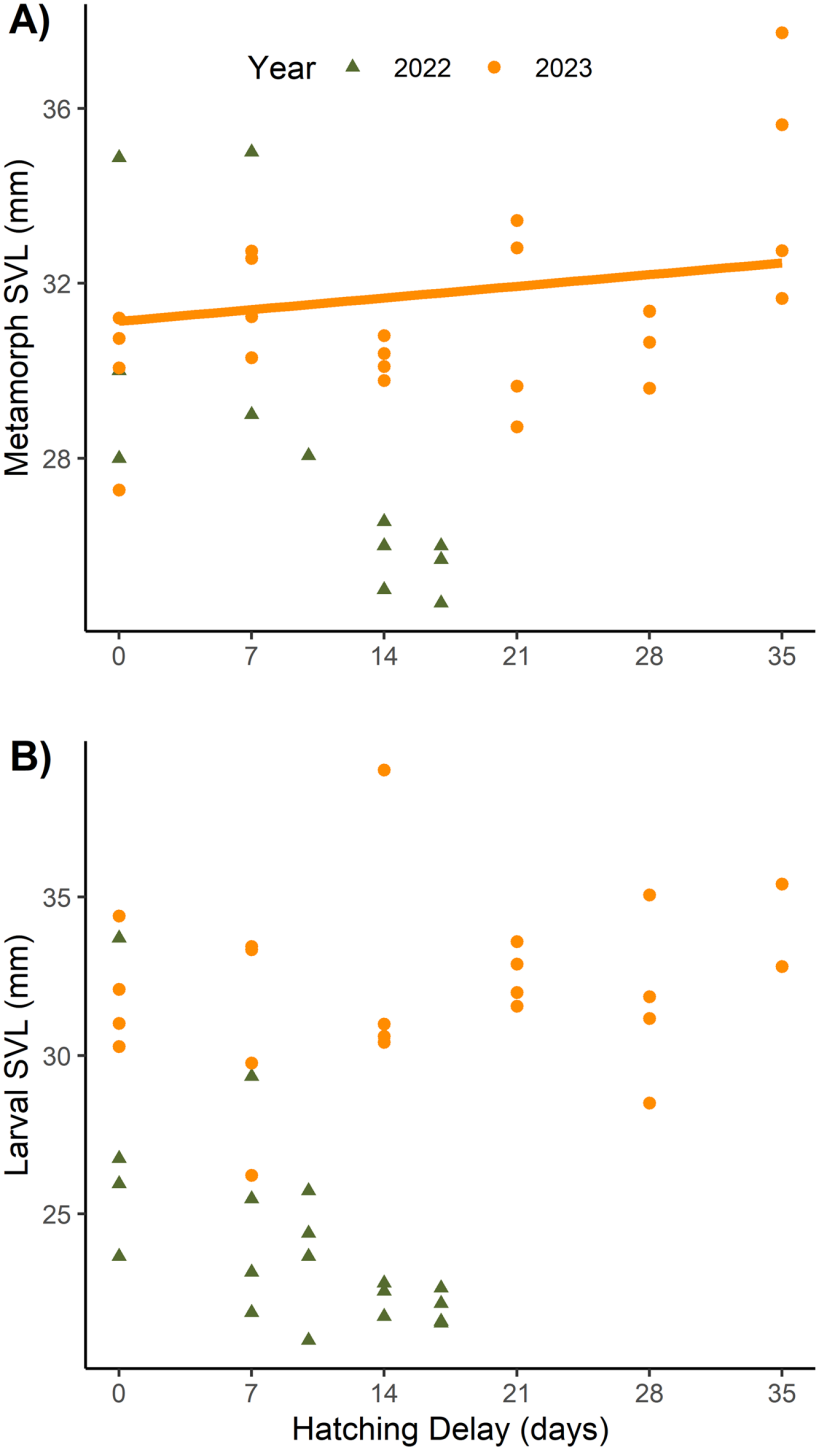
Snout-vent length (SVL, in mm) of surviving metamorphs (**A**) and larvae (**B**) based on hatching delay treatment. The line is the predicted values for SVL at the average survival. Each point represents a tank average. The line in (**A**) shows the predicted values at the average overall survival (Table 2). Lines are absent for 2022 in (**A**) and both years in (**B**) as they represented non-significant relationships (Table 2).

Total survivor number in tanks had a significant, negative effect on snout-vent length (SVL) for metamorphs and larvae across both years (Tables 2, 3; Fig. 2, S1). The quadratic model of hatching delay was more supported over the linear model for both metamorphs (LRT: χ^2^ = 5.50, *P* = 0.03) and larvae (LRT: χ^2^ = 4.67, *P* = 0.03) in 2022. The reverse was true in 2023, where the linear model was more parsimonious for SVL of metamorphs (LRT: χ^2^ = 0.46, *P* = 0.50) and larvae (LRT: χ^2^ = 0.22, *P* = 0.64). After controlling for survivor numbers, hatching delay had a significant positive effect on metamorph SVL in 2023 and was not significant in 2022 (Table 2, Fig. 3a). For larvae, after controlling for survival, individuals tended to be smaller when there was a greater hatching delay in 2022, though these relationships were not statistically significant (Table 3, Fig. 3b). Variability in SVL among larvae in tanks was also higher with a greater hatching delay (Table 3; Fig. S2). Hatching delay did not affect larval SVL in 2023 (Fig. 3b).

**Table 2:** Parameter estimates of linear models for metamorph responses in Q1. Metamorph responses include snout-vent length (SVL) and day of year (DOY) of metamorphosis. For parameters, Delay is the coefficient for the linear term associated with hatching delay treatment, Delay2 is the quadratic coefficient associated with hatching delay treatment, and Survival is the coefficient associated with the effect of number of survivors.

Year	Metamorph response	Parameter	Estimate	SE	*P* value
2022	SVL	Intercept	39.079	1.618	< 0.001
		Survival	− 1.154	0.214	< 0.001
		Delay	− 0.649	0.247	0.030
		Delay2	0.034	0.016	0.075
	DOY	Intercept	158.501	3.188	< 0.001
		Survival	− 0.050	0.464	0.917
		Delay	− 0.023	0.198	0.924
2023	SVL	Intercept	33.251	1.703	< 0.001
		Survival	− 0.387	0.179	0.043
		Delay	0.067	0.032	0.049
	DOY	Intercept	168.621	5.067	< 0.001
		Survival	− 0.108	0.533	0.842
		Delay	− 0.249	0.095	0.016

**Table 3:** Parameter estimates of linear models for larval snout-vent length (SVL) responses in Q1. SVL variability is the coefficient of variation for all surviving larvae in a tank. For parameters, Delay is the coefficient for the linear term associated with hatching delay treatment, Delay2 is the quadratic coefficient associated with hatching delay treatment, and Survival is the coefficient associated with the effect of number of survivors.

Year	Larval response	Parameter	Estimate	SE	*P* value
2022	SVL	Intercept	34.135	1.301	< 0.001
		Survival	− 0.942	0.157	< 0.001
		Delay	− 0.509	0.179	0.015
		Delay2	0.021	0.010	0.059
	SVL Variability	Intercept	0.019	0.027	0.491
		Survival	0.008	0.001	0.596
		Delay	− 0.001	0.004	0.041
2023	SVL	Intercept	37.132	2.409	< 0.001
		Survival	− 0.680	0.268	0.020
		Delay	0.038	0.045	0.403

For day of year (DOY) of metamorphosis, LRT indicated the linear model was supported over the quadratic model for the duration of hatching delay (2022: χ^2^ = 0.39, *P* = 0.53; 2023: χ^2^ = 0.76, *P* = 0.38). Survival did not affect DOY of metamorphosis in either year, and hatching delay had a significant negative effect only in 2023 (Table 2; Fig. S3).

### Question 2: Seasonality of hatching delay

**Figure 4:**
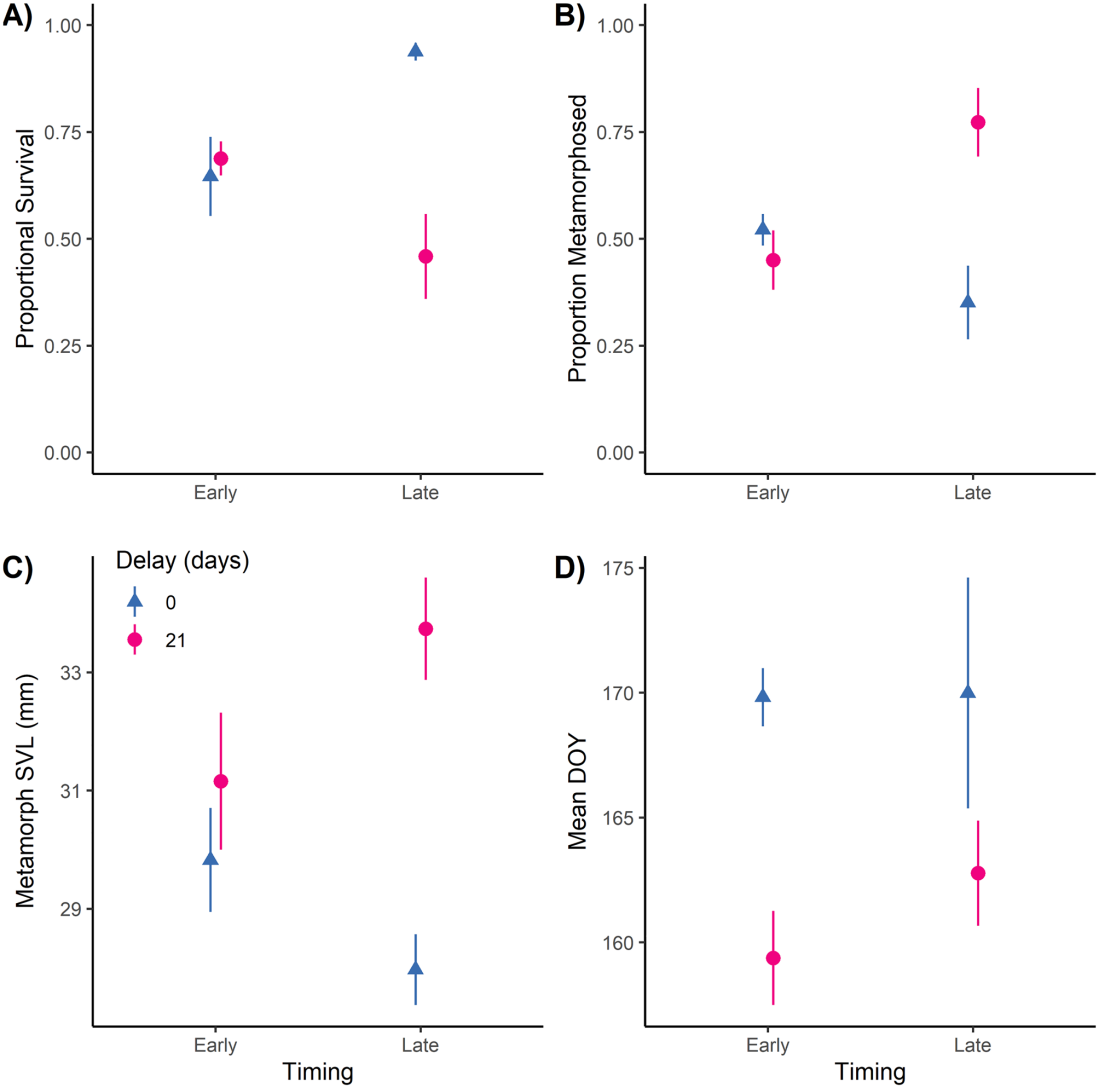
Proportional survival (**A**), proportion of survivors that metamorphosed (**B**), mean snout-vent length (SVL, in mm) of surviving metamorphs (**C**), and mean day of year (DOY) of metamorphosis (**D**) in the hatching delay-seasonality experiment (Q2). Colors and shapes represent hatching delay treatment (no delay = blue triangles; 21-day delay = pink circles) with early and late seasonality treatments (x-axis). All points are means ± SE. There was a significant interaction for survival in (**A**); once survival was accounted for in other panels, no treatment differences were significant.

There was a significant interaction between seasonality and duration of hatching delay for *A. texanum* survival (χ^2^ = 18.36, *P* < 0.001). Survival was similar regardless of the duration of hatching delay (~ 70%) for the early seasonality, but was nearly twice as high with no delay (94%) compared to a 21-d delay (46%) in the late seasonality (Fig. 4a). The proportion of survivors that achieved metamorphosis also showed a significant interaction of seasonality and duration (χ^2^ = 5.98; *P* = 0.01), but with the 21-d delay having higher rates of metamorphosis in the late seasonality treatment; no differences were apparent in the early seasonality treatment (Fig. 4b).

Final SVL of metamorphs and larvae were negatively related to the number of survivors in a tank (metamorphs: F_1,11_ = 9.84, *P* = 0.009; larvae: F_1,11_ = 19.82, *P* = 0.001). After controlling for survival, the interaction of seasonality and hatching delay was not significant for the models of metamorph and larval SVL (metamorphs: F_1,11_ =  = 0.38, *P* = 0.55; larvae: F_1,11_ = 0.00001, *P* = 0.99), and was thus removed from those models. Hatching delay had a marginal effect on metamorph SVL (F_1,12_ = 4.31, *P* = 0.06; Fig. 4c), where metamorphs were larger with a 21-d hatching delay. Seasonality was not significant for metamorph SVL (F_1,12_ = 0.51, *P* = 0.49). There were no significant treatment differences in larval size for either hatching delay (F_1,12_ = 1.37, *P* = 0.26) or seasonality (F_1,12_ = 0.35, *P* = 0.56) after controlling for survival. Metamorphosis DOY was not affected by survival (F_1,11_ 0.37, *P* = 0.56) or the interaction of seasonality and hatching delay (F_1,12_ 0.34, *P* = 0.57, and thus those variables were not retained in that model. There was a significant effect of hatching delay (F_1,13_ 10.72, *P* = 0.006), where DOY of metamorphosis was approximately 10 d earlier in the 21-d treatment compared to the 0-d delay treatment (Fig. 4d). Seasonality did not affect DOY of metamorphosis (F_1,13_ 0.44, *P* = 0.52).

### Hatchling size

Hatchling total length (TL) in 2022 was significantly different across the hatching delay treatments (F_4,230_ = 21.50, *P* < 0.001), with most of the differences occurring between the first addition and the remaining additions (Fig. S4, Table S2). In 2023, hatchling TL was also different across addition dates (F_5,264_ = 28.56, *P* < 0.001), but in a different pattern. In this year, the first and last additions was smaller than the middle additions (Fig. S4, Table S2).

### Water temperature

Water temperatures were similar across years in terms of daily averages and variability, though 2022 temperatures were on average lower for the same day of year, especially early in each experiment (Fig. S5). Water temperate was approximately 1.3 degrees C warmer in the late seasonality treatment compared to the early seasonality treatment (χ^2^ = 39.02, *P* < 0.001). This was largely driven by the difference early on in the experiment, where the first two weeks of each treatment were approximately 4 degrees C different on average (χ^2^ = 179.45, *P* < 0.001).

## Discussion

Overall, we found that intraspecific priority effects can influence demographic responses but that the outcomes were contingent on the exact way in which hatching delays occurred. First, demographic traits showed a combination of linear and non-linear responses to hatching delays across cohorts, with the strongest effect (e.g., most reduced survival) occurring at the longer duration treatments. Second, we found that the timing of *when* hatching delays occur matters; in our case, hatching delays shifted to later in the season had a stronger effect on demographic responses than when delays occurred earlier in the season, resulting in stronger priority effects in the former. Collectively, these results indicate much nuance to intraspecific phenological variation in its impacts on demography, which may complicate our understanding of intraspecific shifts in phenology in relation to climate change.

The duration of hatching delay between cohorts affected several demographic traits in our study in a non-linear fashion. At longer durations (> 28 days) between cohort additions, survival and metamorphosis rates decreased and increased, respectively. This outcome was likely induced by either strong size-structured competition that led to indirect mortality and/or cannibalism across cohorts that reduced survival via direct mortality. Untangling which mechanism prevailed was not possible given our design because we could not track individuals or cohorts, but predation tends to result in stronger mortality in salamanders than resource limitation^26^. We presume the 44% mean survival at 35-d were largely made up of the 4 early individuals, as seen in other studies^20,21^. The non-linear response of metamorphosis rates of survivors in 2023 was likely driven by the non-linear survival response: competition was reduced at lower survival, increasing the likelihood individuals could achieve the size necessary for metamorphosis.

For the short hatching delay treatments, we interestingly saw an increase in survival (~ 12% increase on average from 0 to 14 days delay), resulting in the observed the non-linear relationship. The increase in survival across short delay treatments was consistent across both years’ experiments, despite some differences in experimental set-up (see Methods). One possible explanation is resource partitioning by larvae of different size classes. When cohorts were added more synchronously (0–7 days), then exploitative competition may have increased due to greater larval body size similarities, reducing survival. When they were added less synchronously (> 21 days), then interference competition and/or cannibalism likely increased, again decreasing survival by an even larger amount (~ 34%) than the initial increase in survival at 14 days. As stated above, we unfortunately could not track individuals or cohorts, so we cannot fully evaluate their intraspecific interactions. Overall our results do show that both greater and lesser hatching synchrony could negatively impact populations. Such nonlinear demographic responses to phenological shifts in general have been underexplored in many taxa^27^, and should be considered further to understand both their prevalence and/or importance.

The hatching delay treatments had less consistent effects on other demographic traits like body size or the date of metamorphosis, and were more affected by density dependence (i.e., the number of survivors), a pattern consistent with numerous other studies^28,29^. We also found strong evidence for compensatory growth, whereby individuals that are delayed catch up in development or growth^30^. In our case, individuals metamorphosed on average at the same time and size from the no-delay treatments in Q2, despite a two-week delay in hatch timing. Thus, being delayed in hatching phenology did not incur life history costs, which matches other studies of compensatory growth in amphibians^31,32^. At the same time, we could not track many individuals through metamorphosis in 2022 due to early termination of the experiment (see Methods) so our inference about metamorph responses–a key metric of future fitness potential^33^–are limited from that year.

Initial hatchling size varied among our addition treatments, which could have impacted some of our results. For instance, mortality in the 35-d treatment may have been higher due to the later hatchlings being on average smaller than earlier additions, making them easier to consume. Additionally, the intermediate duration treatments in 2023 may have had lower predation due to those hatchlings being slightly larger on average. Attributing hatchling sizes to rearing conditions is further complicated by eggs not starting at the same development stages (especially in 2022)—eggs that developed more under more natural conditions for several days experienced different environmental conditions than those entirely reared in the lab, which could have impacted their size at hatching. Other work on salamanders shows patterns opposite to ours: eggs that experience cooling and/or cooler temperatures during development typically hatch larger^26,34,35^. Therefore, further investigations into how temperature regimes that eggs experience during development affect traits like size at hatching could help pinpoint impacts on experimental outcomes, as well as more generally how climate change may affect demographic traits of organisms based on temperatures they experience^5^.

Demographic responses were affected by the seasonality of when hatching delays occurred in our experiments. These results were largely dictated by how seasonality and hatching delay treatments impacted survival: when delays occurred later in the season, survival was substantially reduced, resulting in body size increases of survivors due to reduced intraspecific competition. While we do not know the precise mechanism behind these results, one potential mechanism is temperature differences across the treatments: on average, larvae in the late season priority effects treatment experienced water temperatures ~ 1.5 degrees warmer than our early season priority effects treatment. This difference was even greater early on in each treatment, where temperature was ~ 4 degrees C colder in the early season treatment. Such colder temperatures may have suppressed activity levels and/or growth rates of larvae in the early season treatment, which would have limited the ability of larvae to grow sufficiently to then consume the delayed individuals. Alternatively, the later additions may have been able to grow faster under warmer water conditions to exceed the gape of the stunted earlier-arrived individuals in the early seasonality treatment, increasing survival. In contrast, the late seasonality treatment all occurred under relatively warmer conditions, resulting in presumed higher rates of cannibalism. However, because we did not factorially manipulate temperature along with hatching phenologies (i.e., they occurred simultaneously), as in other studies^10^, our results should be interpreted with caution. A controlled lab study that manipulated temperature or other seasonally changing variables independent of priority effects would further unravel the mechanisms behind such processes. In other ecological systems, temperature was shown to modify the strength of priority effects in some cases^8,13,24^, but not others^10^. Of course, temperature in our study is also confounded with other variables that differ seasonally, such as day length, which could also have influenced these results. Thus, greater investigation into how seasonal shifts in phenological distributions, and the associated abiotic changes (e.g. temperature variation) organisms experience with such overall shifts, are needed to unravel how priority effects may differ within or across seasons.

Priority effects generated through hatching asynchronies have shown pronounced negative effects on later arriving individuals in some other studies of amphibians that used similar durations to our longest treatment differences (e.g., ~ 40 days)^12,20^, whereas other studies have found more substantial intra- or interspecific priority effects at shorter durations^13,14,21,36,37^. Why effect sizes differ to such a degree across studies is not entirely known, but could be due to intrinsic species traits or external environmental conditions. For example, food levels within experimental venues may differ enough that would permit satiation of early-arriving individuals, leading to reduced magnitudes of priority effects; in our study, this manifested as reduced cannibalism. Individual species behaviors may promote greater/lesser impacts on subsequently arriving individuals/species. In our case, our focal species may simply not be that cannibalistic, and had we chosen a species with more voracious foraging abilities or cannibalistic phenotypes like *A. tigrinum* or *Hynobius retardatus*^21,38^, our results may have been more pronounced.

Our experiments moreover have important practical implications for further experimental tests aimed at understanding priority effects, as well as climate-driven phenological shifts. The timing of when experiments are conducted seasonally, as well as the range of phenological manipulations (in our case, the duration of a priority effect), may greatly impact the conclusions drawn from such studies. Had we only conducted the mesocosm study in 2022 with a range of 17-d difference it would have resulted in very different conclusions regarding the importance of priority effects than the two years of studies combined. Similarly, the time periods in which we conducted Q2 resulted in different conclusions about the importance of priority effects; had we only conducted an early-season experiment, we would have found that hatching delays did not matter. Furthermore, both 14-d hatching delay treatments in 2022 and 2023 resulted in similar conclusions in terms of survival, despite being set up across different time periods (2022: April 4 and 18; 2023: March 16 and 30). The temperature ranges these treatments experienced were in fact more similar than treatments set up in April across the two years (i.e., early April 2022 was more similar to mid-March 2023 in temperature than early April 2023). Investigators are often constrained by when seasonally active species appear, and thus are sometimes limited in the degree to which phenology can be mimicked or altered, at least in outdoor mesocosms like ours. However, such design details, whether forced by animal life histories or by experimenter choices, must be considered when evaluating the consequences of phenological shifts.

Phenological shifts are well-documented at this point, with the next step forward for many systems being further understanding of the importance of such changes. Overall, our study demonstrates that how important phenological shifts and priority effects are for different taxa likely will depend on the context of such shifts. For example, earlier breeding is a common response by spring-breeding amphibians to climate change^39,40^, which based on our results would suggest priority effects may actually decrease in strength because the developing larvae often experience colder water temperatures during development after breeding earlier in the season^5^. Additionally, the degree of breeding asynchrony *and* synchrony induced by climate change may both profoundly affect the strength of priority effects, as evidenced by our nonlinear survival response to hatching delays. Studies therefore need to match such realties of nature, with greater investigation of gradients of phenological shifts across a variety of conditions e.g.,^27^. Additionally, an overall shift of organisms’ phenological distributions to where intra- and interspecific interactions unfold under different abiotic conditions, as our seasonality manipulation of priority effects demonstrated, may alter how those interactions proceed.

## Materials and Methods

### Study organism

*Ambystoma texanum* is commonly found in the central United States (Petranka 1998). Adults undergo breeding migrations in the early spring to ponds and wetlands, where eggs are deposited. Larval development typically takes 2–4 months, and metamorphosis occurs after individuals exceed 17 mm snout-vent length (SVL). Within a given population in this species, variation in the timing of breeding is common; for instance, in spring 2022 when part of this study was conducted, at least 3 breeding pulses were observed to occur (15 February, 18 March and 1 April) in ponds nearby to where the experiment was conducted (personal observation). Additionally, egg placement varied in depth from 50 cm in the direct center of the pond to about ~ 10 cm of water on the edge of the pond (personal observation), which can influence hatching rates due to different temperature regimes. After hatching, larval diet consists of small invertebrates like zooplankton or dipteran larvae (Bachmann, Smith and Petranka 1987). Cannibalism has not been documented in this species, but is likely given the body size structure that can develop in ponds and this interaction’s general prevalence within the *Ambystoma* genus^20,38,41,42^.

### Experimental design

To address our research questions, we conducted a total of three experiments across two years. To address Q1, we conducted similarly designed experiments in 2022 and 2023 that manipulated the relative timing of hatching in *A. texanum*, such that two cohort additions (N = 4 and N = 8 hatchlings, respectively) were spaced out over different temporal durations. We used unequal densities across our two additions to promote growth by the early cohort that would potentially amplify effects on the later cohorts^43^. In 2022, we added our two cohorts 7, 11, 14 and 17 days apart, with four hatchlings added on Day 1 and eight hatchlings added on the second date (Table S1). Because we saw limited treatment differences in most responses for these introduction timings, we repeated the same basic design of the experiment in 2023, but with greater temporal separation: 7, 14, 21, 28 and 35-days difference in addition between the two cohorts (Table S1), to better understand whether there was an upper limit which demography may be altered by hatching asynchronies. The temporal durations of hatching delays used across both experiments are generally consistent with observed variation in breeding in phenology of *A. texanum*. The 35-d separation treatment would represent large variation in time between pulses, but would be consistent with hypothesized increasing variability in rainfall with climate change. In both experiments, we also had a no delay control, where all 12 hatchlings were added at the same time to each tank on Day 1 of the respective experiments. Each treatment in both experiments was replicated four times. Larval densities (26/m^2^) were at or exceeded those used by other researchers in similar priority effect studies^12,20^, but matched natural densities we have observed (unpublished data).

To address Question 2, we conducted a simultaneous 2 × 2 experiment in 2023 that crossed seasonality (early or late) with temporal duration (0 or 21-day delay in addition). The early season 21-d delay treatments had individuals added on 16 March and 6 April, whereas hatchlings in the late season delay were added on 30 March and 20 April, creating a 14-day temporal delay for the seasonality component. For the temporal duration treatments, 12 hatchlings were added on the same day (0-day) or split across 21 days (N = 4 and N = 8 on each date, respectively). Each treatment had four replicates.

### Experimental set-up

All experiments were conducted outdoors in 100 l water tanks (Tuff Stuff; 76 cm diameter, 35 cm height) at a secure facility on the Southern Illinois University Edwardsville (SIUE) campus. All experiments were conducted with SIUE Institutional Animal Care and Use Committee approval (#992), and in accordance with relevant guidelines and regulations including the ARRIVE guidelines (https://arriveguidelines.org). Tanks were filled to a depth of ~ 26 cm, and had small drain holes drilled in the sides just above the water level to prevent overflow. Hose water was added as needed to maintain constant water levels. Leaves (primarily oak [*Quercus sp.*]) were added shortly after filling, along with concentrated aliquots of plankton to form the base of the food web. Tanks were covered with 70% shade cloth covers to limit colonization by predatory insects. In 2022, gray tree frogs (*Hyla versicolor*) were able to crawl under the lids in two tanks and lay eggs. The subsequent tadpoles were haphazardly redistributed amongst all tanks so that all replicates had at least some tadpoles, though the number of tadpoles per tank was not equal. These tadpoles served as additional prey items for the salamanders. No colonization by gray tree frogs occurred in 2023.

We collected salamander eggs from ponds on the SIUE campus with permission from the Illinois Department of Natural Resources (HSCP 22–07 and HSCP 23–08). Collections were made from ponds that are known to dry prior to metamorphosis in *A. texanum*, limiting any population impacts on this species. In 2022, eggs were collected once and were at varying stages of development: early (Harrison stages 1–10), middle (Harrison stage: 25–30) and late (Harrison stage > 38), representing at least two distinct breeding events (Table S1). Eggs at Harrison stages 25–30 were likely the first breeding cohort but had been placed in relatively deeper colder water and thus were delayed in their development. Eggs were transported back to an indoor lab space, where they were divided into different containers based on development stage. The middle and late stage eggs were placed on a lab table top under a light set to mimic the natural photoperiod (~ 12:12). Water temperature was a constant 18 degrees C. The early stage eggs were subdivided into three containers, with one container being placed on the lab table and two being placed inside an environmental incubator set to a constant 10 degrees C and 12:12 light:dark cycle. Aged tap water was used to exchange 50% of the water every 5 days in each container to ensure oxygenated conditions. We removed the two containers from the incubator after 5 and 10 days, respectively, and placed on the lab table to permit hatching. In 2023, eggs were collected on multiple collection dates, with most eggs at early Harrison stages (1–10; Table S1). Hatching was delayed as above by incubating them at 6 C and 12:12 light:dark cycle. We removed eggs from the incubator approximately one week prior to their addition date to ensure hatching occurred. After hatching, we photographed hatchlings in a pan of water with a ruler before being transferred to the experimental tanks. Total length (TL) of hatchlings was obtained from these photographs using ImageJ (Rasband 1997).

We monitored tanks for signs of metamorphosis starting in mid-May of each year, which matches the typical end of the larval period in this species^44^. Metamorphs were removed after they were found to prevent drowning, and SVL measured by hand using a ruler (2022) or through photographs in ImageJ (2023). In 2023, wet mass (nearest 0.01 g) was also recorded using a ProScout scale (Ohaus). At the conclusion of each experiment, tanks were drained and the leaf litter carefully searched for all remaining individuals. Remaining individuals (larvae and metamorphs) were then photographed above a ruler to again measure SVL using ImageJ (Rasband 1997). We calculated survival as the number of surviving larvae + metamorphosed animals. Early termination (i.e., before all individuals completed metamorphosis) occurred in each year because of excessive heat (air temps > 38 C for multiple days) that would have likely resulted in substantial heat-related mortality of larval salamanders. In 2022, we terminated the experiment on 10 June (Day 67), shortly after metamorphosis began (9% of individuals). In 2023, the experiment was terminated on 28 June (Day 104), at which point 48% of individuals had completed metamorphosis.

### Analysis

All analyses were conducted using R^45^. For Question 1, survival across both experiments was analyzed using generalized linear models with a binomial error distribution^46^. We checked for overdispersion but none was present in the models. Linear and quadratic terms of hatching delay (i.e., the number of days from the start of the experiment prior to each addition) were included as covariates. We modeled each year separately due to the differences in experimental procedures (e.g., duration of experiment, presence of tree frog tadpoles in 2022). We compared these models to ones that only included the linear term for hatching delay duration using likelihood ratio tests. We then interpreted significance of the coefficients in the most supported models to examine relationships with survival. We similarly analyzed whether hatching delay was related to the metamorphosis rates, though we examined different response variables in each year because of the overall experimental duration differences. For the 2022 experiment, we analyzed the occurrence of metamorphosis in a tank (as only 60% of tanks had at least one metamorph) to determine if hatching delay resulted in the initiation of metamorphosis. In 2023, we analyzed the proportion of the survivors that completed metamorphosis, as the overall experimental duration was longer, resulting in more metamorphosis. In both models, we used the same covariates and linear vs quadratic model comparison as above.

For individual traits in Q1, we analyzed tank averages for SVL of both metamorphs and larvae, and tank-averaged day of year (DOY) of metamorphosis. We do not know the true larval period duration for any individuals in treatments where the cohorts were added asynchronously, i.e., we only know the true larval duration in the 0-day control, as it was infeasible to track individuals and the cohorts did not maintain distinct size categories as in other studies^14^. By analyzing day of year of metamorphosis, we could still assess the degree of synchronization of metamorphosis across the hatching delay treatments. For all response variables, we used linear models with hatching delay as the primary covariate. We also included the total number of survivors for a given tank as an additional covariate, as the surviving density often has significant effects on individual life history traits via density dependence. For 2022, we also analyzed variation in larval body size at the end of the experiment, calculated as the coefficient of variation (CV) in SVL, again with hatching delay and total survival as covariates. This analysis allowed us some insight into whether we could see distinct size classes within the remaining individuals that mirrored our priority effect treatments, where asynchronous treatments were expected to have higher CV values.

For Q2, we again analyzed average SVL of larvae and metamorphs, DOY of metamorphosis, proportion metamorphosed, and survival. We used analysis of variance (ANOVA) for morphometric data and generalized linear models (quasibinomial errors to correct for overdispersion) for survival and proportion metamorphosed, but for predictors we included the categorical variables of seasonality, hatching delay duration, and their interaction. Significance of each term was based on F-tests or chi-squared statistics, as determined by the *Anova* function in the `car` package^47^. Whenever treatment differences were significant, we used Tukey post hoc tests from the `emmeans` package to determine which pairwise comparisons were statistically different^48^.

For both experiments, hatchling TL was compared across treatments using ANOVA. We conducted this analysis for each year separately. We then conducted Tukey post hoc tests to compare which hatching delay addition treatments (i.e., weeks) were different from each other.

We analyzed water temperature across the experiment in two ways. First, we compared average temperature across the duration of the early and late seasonality treatments in Q2 using linear mixed models, with tank as a random effect to account for non-independence of measurements. We also compared temperature over the first two weeks of each seasonality treatment, with the expectation that this time period would drive overall differences, as well as impact growth trajectories of larvae.

### Supplementary Information

Supplementary Information.

## Data Availability

Data is available in the Zenodo repository at 10.5281/zenodo.10636154.
