# Supplementary material for: Intraspecific priority effects in response to egg hatching delay in a pond-breeding salamander
Source: Sci Rep. 2024 Aug 21;14:19436. doi: 10.1038/s41598-024-70140-z (PMC11339462; doi:10.1038/s41598-024-70140-z)
Supplement: Supplementary file 1 — Supplementary Information. [file 41598_2024_70140_MOESM1_ESM.pdf]

Supporting Information

Title: Intraspecific priority effects in response to egg hatching delay in a pond-breeding salamander

Author: Thomas L. Anderson* and Trevor J. Rallo

Affiliation: Department of Biological Sciences, Southern Illinois University Edwardsville, Box 1651, Edwardsville, IL 62026

* corresponding author:

Table S1: Hatching delay treatments, dates of experimental additions, Harrison development stages of eggs when collected and the numbers of days eggs were delayed in the environmental chamber across the two years of experiments. No “Date 2” exists for the 0-d hatching delay treatment, as all individuals were added on the same day.

Year	Hatching Delay Duration (d)	Date 1	Date 2	Stage	Days Delayed
2022	0	5 April	—	> 38	0
	7	5 April	11 April	25-30	0
	11	5 April	14 April	1-10	0
	14	5 April	17 April	1-10	5
	17	5 April	21 April	1-10	10
2023	0	16 March	—	>38	0
	7	16 March	22 March	1-10	0
	14	16 March	29 March	1-10	7
	21	16 March	6 April	1-10	14
	28	16 March	13 April	1-10	21
	35	16 March	20 April	1-10	28

Table S2: Tukey HSD pairwise contrasts of hatching delay treatments for hatchling total length. The contrast column indicates which addition dates are being compared. Bold values indicate significantly different contrasts.

Year	Contrast	Difference	Lower	Upper	P-value
2022	**7-0**	**-0.724**	**-1.162**	**-0.287**	**0.000**
	**11-0**	**-1.300**	**-1.743**	**-0.857**	**0.000**
	**14-0**	**-0.768**	**-1.205**	**-0.330**	**0.000**
	**17-0**	**-0.809**	**-1.247**	**-0.372**	**0.000**
	**11-7**	**-0.576**	**-1.123**	**-0.028**	**0.034**
	14-7	-0.043	-0.587	0.500	0.999
	17-7	-0.085	-0.628	0.458	0.993
	14-11	0.532	-0.016	1.080	0.061
	17-11	0.490	-0.057	1.038	0.103
	17-14	-0.042	-0.585	0.502	1.000
2023	**7-0**	**1.194**	**0.542**	**1.846**	**0.000**
	**14-0**	**0.994**	**0.449**	**1.539**	**0.000**
	**21-0**	**0.867**	**0.215**	**1.519**	**0.002**
	28-0	0.623	-0.029	1.275	0.070
	**35-0**	**-0.643**	**-1.199**	**-0.088**	**0.013**
	14-7	-0.200	-0.782	0.382	0.922
	21-7	-0.327	-1.011	0.356	0.742
	28-7	-0.571	-1.254	0.113	0.161
	**35-7**	**-1.837**	**-2.429**	**-1.245**	**0.000**
	21-14	-0.127	-0.710	0.455	0.989
	28-14	-0.371	-0.953	0.211	0.448
	**35-14**	**-1.637**	**-2.109**	**-1.166**	**0.000**
	28-21	-0.244	-0.927	0.440	0.910
	**35-21**	**-1.510**	**-2.102**	**-0.918**	**0.000**
	**35-28**	**-1.266**	**-1.858**	**-0.674**	**0.000**

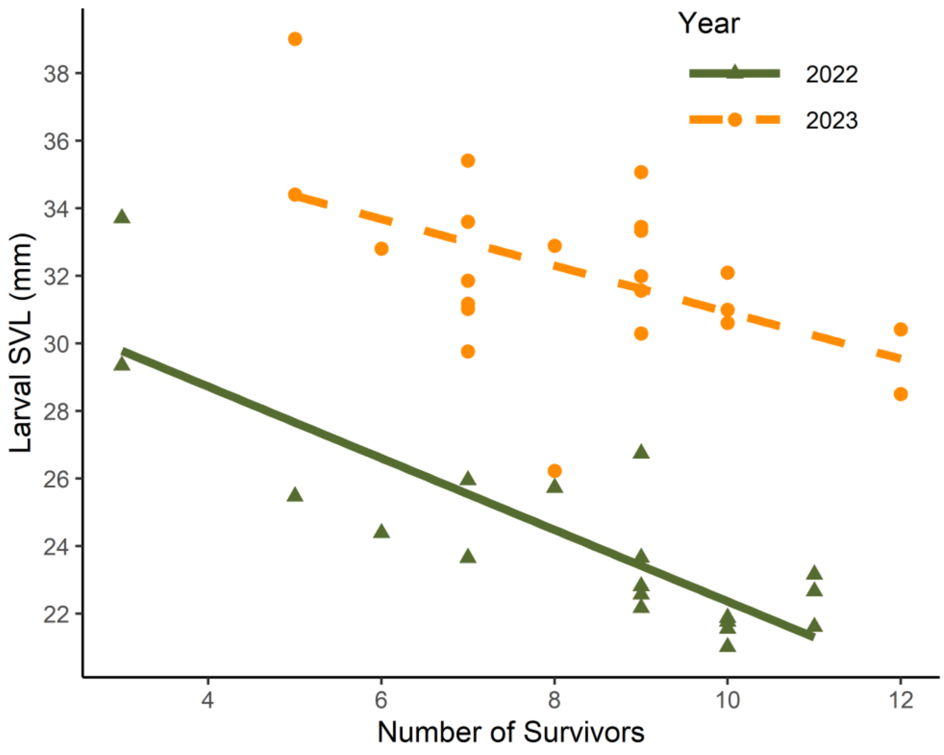

Figure S1: Snout-vent length (mm) of larval *A. texanum* in response to total number of survivors in a tank. Each point represents at tank average. Point shape and line type indicates year. Models were fit separately for each year.

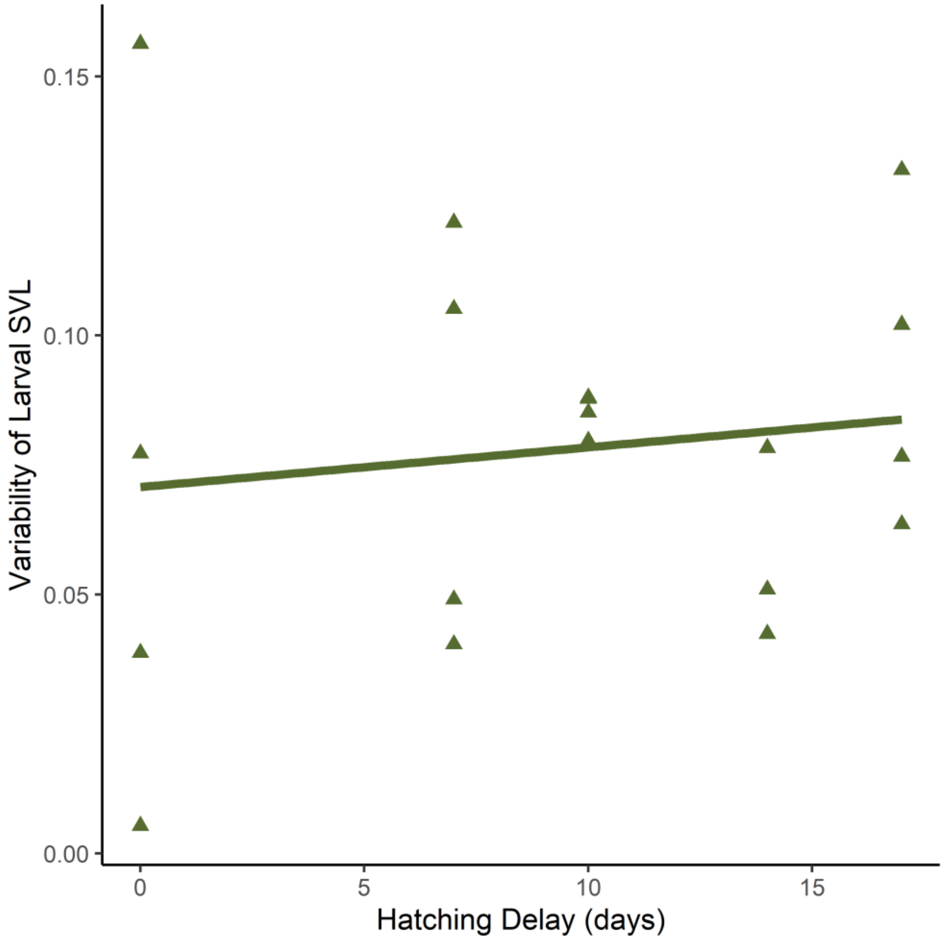

Figure S2: Variability (coefficient of variation) in snout-vent length (SVL) of larval individuals at the termination of the experiment in response to hatching delay in 2022. The relationship was significant after controlling for survival (Table S1). Analysis of variability was not performed in 2023 due to more individuals completing metamorphosis in that year.

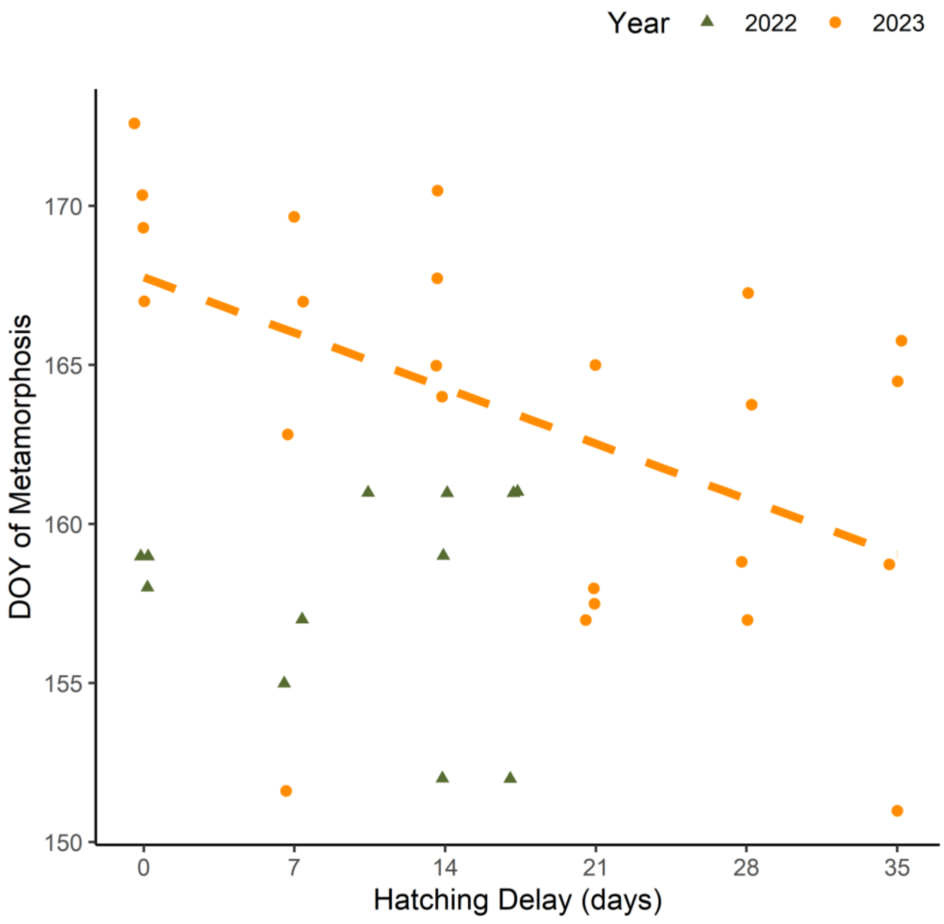

Figure S3: Average day of year (DOY) of metamorphosis for tanks across the hatching delay treatments. Point shape and color indicates year. The model was significant for only 2023.

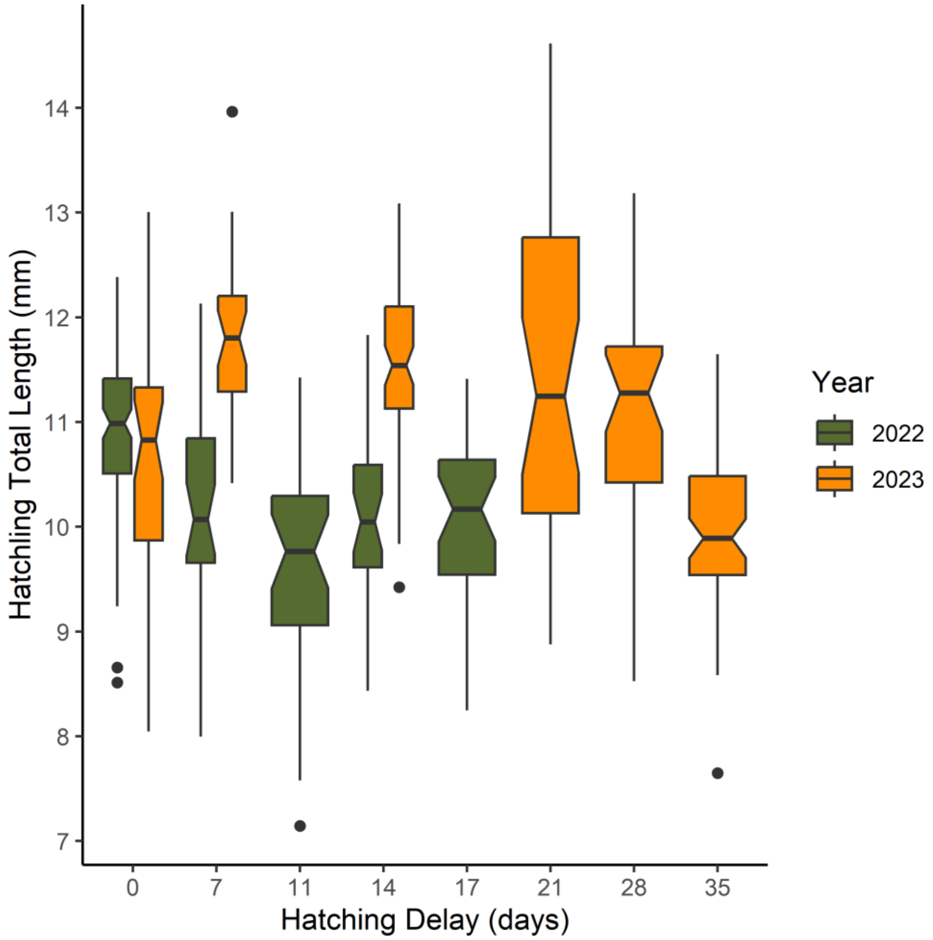

Figure S4: Hatchling total length (in mm) for the different hatching delay treatments in 2022 (green) and 2023 (orange). Within a year, boxes with notches that do not overlap would suggest significant differences. For complete treatment differences, see Table S3.

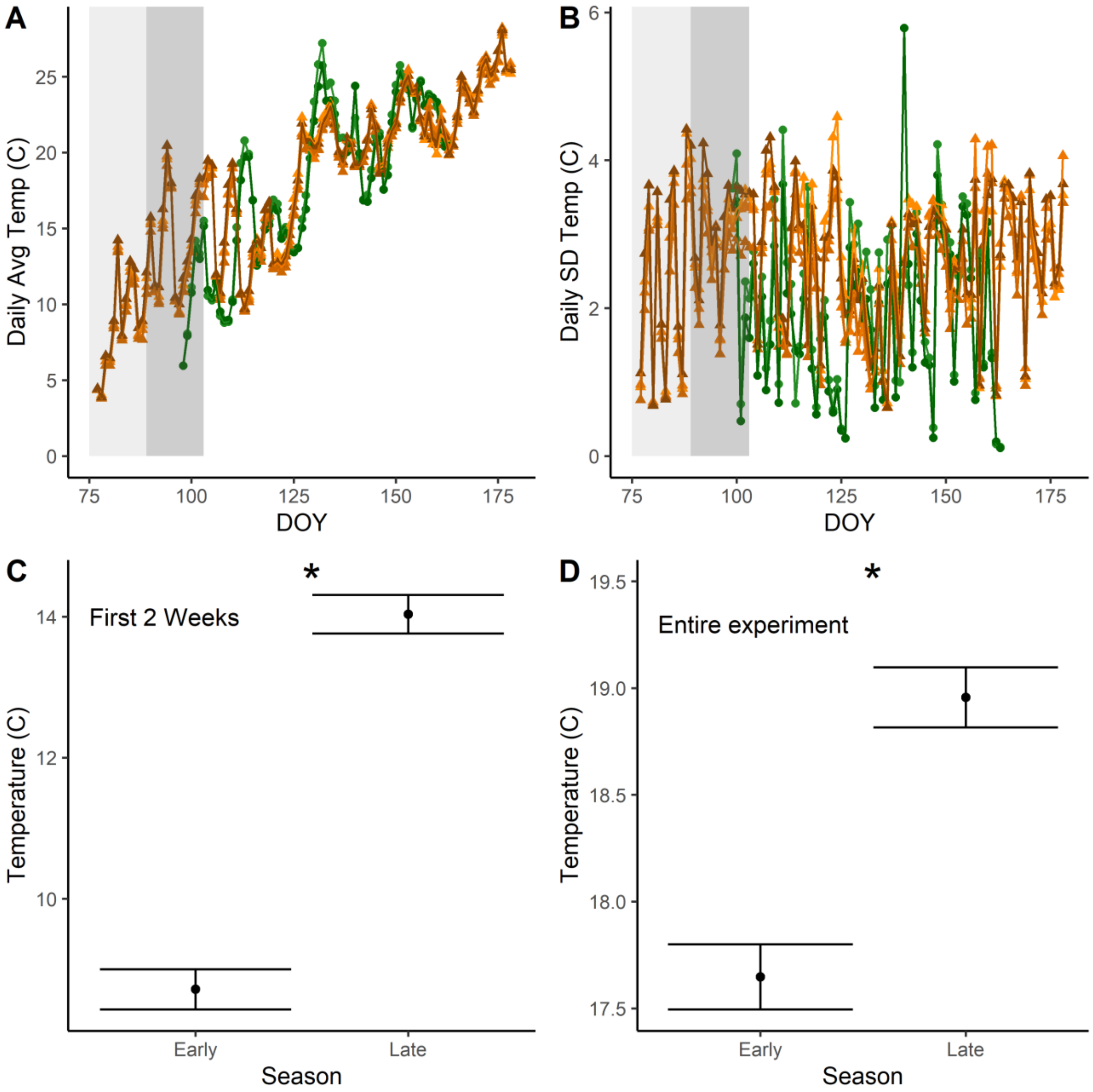

Figure S5: Water temperature profiles of mesocosms across the two years of experiments (N = 2 loggers in 2022; N = 4 loggers in 2023). Panels A and B show the daily average and standard deviation in temperature. Green colors and circles indicate tanks in 2022, and orange colors and triangle shapes indicate tanks in 2023. Panel C shows the mean (± SE) water temperature of tanks for the first two weeks of the early (light gray shaded area of Panels A and B) and late (dark gray shaded area of Panels A and B) seasonality treatments in 2023. Panel D shows the overall average temperature of the same treatments across the entirety of the experiment. * indicates both comparisons were significantly different in Panels C and D.
